# Pupil Response and the Subliminal Mere Exposure Effect

**DOI:** 10.1371/journal.pone.0090670

**Published:** 2014-02-28

**Authors:** Sanae Yoshimoto, Hisato Imai, Makio Kashino, Tatsuto Takeuchi

**Affiliations:** 1 Department of Psychology, Japan Women’s University, Kawasaki, Kanagawa, Japan; 2 NTT Communication Science Laboratories, NTT Corporation, Atsugi, Kanagawa, Japan; 3 CREST, Japan Science and Technology Agency, Atsugi, Kanagawa, Japan; 4 Department of Psychology, Gakushuin University, Toshima-ku, Tokyo, Japan; Monash University, Australia

## Abstract

The subliminal mere exposure effect (SMEE) is the phenomenon wherein people tend to prefer patterns they have repeatedly observed without consciously identifying them. One popular explanation for the SMEE is that perceptual fluency within exposed patterns is misattributed to a feeling of preference for those patterns. Assuming that perceptual fluency is negatively correlated with the amount of mental effort needed to analyze perceptual aspects of incoming stimuli, pupil diameter should associate with SMEE strength since the former is known to reflect mental effort. To examine this hypothesis, we measured participants’ pupil diameter during exposure to subthreshold stimuli. Following exposure, a preference test was administered. Average pupil diameter throughout exposure was smaller when the SMEE was induced than when the SMEE was not induced. This supports the hypothesis that increasing perceptual fluency during mere exposure modulates autonomic nervous responses, such as pupil diameter, and eventually leads to preference.

## Introduction

Preferences for an item or person can be formed by “mere exposure.” This means that simply after repeated observation, we tend to experience a feeling of preference for repeated information [1], [2]. Furthermore, Kunst-Wilson and Zajonc [3] showed that people tend to prefer stimuli to which they have been repeatedly exposed subliminally. In their experiment, each visual pattern was presented for only 1 ms; thus, the visual pattern was considered subliminal or below participants’ detection threshold. After observing the visual pattern stimuli, participants were asked to select their preferred pattern from two candidates: one they had previously observed subliminally and the other that they had not observed. Surprisingly, even though participants could not correctly identify which pattern had been presented beforehand, they tended to select the subliminally presented patterns as the preferred one. This is referred to as the “subliminal mere exposure effect (SMEE),” a phenomenon that has been widely replicated [4]–[7].

Although a number of studies assessing the SMEE have been published over the past three decades, existing research has not yet established whether any psychophysiological processes are associated with this effect. In an attempt to fill this gap, we examined the possibility of a relationship between SMEE and pupil response, one of the psychophysiological responses governed by the autonomic nervous system [8].

One popular hypothesis regarding the mere exposure effect under both supraliminal and subliminal conditions is based on perceptual (or cognitive) fluency [9]–[11]. Perceptual fluency refers to the ease of processing incoming stimuli based on manipulations of perceptual quality [12]. Through past experience, such as mere exposure, a stimulus is likely to be processed more fluently. According to this hypothesis, people unconsciously misattribute their perceptual fluency, typically enhanced by mere exposure to a stimulus, to a feeling of preference. In other words, people tend to prefer stimuli they can process fluently. In addition, other hypotheses regarding SMEE that do not include a misattribution process, but an emergence of positive affect toward the exposed stimuli, also assume perceptually fluent processing of those stimuli [10], [13], [14]. Oppenheimer [12] reviewed studies regarding perceptual fluency as a determinant of preference judgments.

Although rigorously defining perceptual fluency is difficult [12], several psychophysical measures, such as the time needed to judge perceptual aspects of visual patterns or the readability of words, have been used to evaluate perceptual fluency in visual information processing [15], [16]. For example, Winkielman et al. [16] showed that when the reaction time to categorize a visual pattern is lower, participants tend to prefer that pattern. They argued that visual processing of the pattern is more fluent when reaction time is lower.

Further, pupil responses are modulated by not only ambient luminance levels but also task difficulty [17]–[21]. These studies have established that a difficult task induces pupil dilation and vice versa. For example, Kahneman and Beatty [17] found that pupil size during a short-term memory task was positively correlated with task difficulty. Using a visual search task, Porter et al. [19] showed a positive correlation between pupil size and search difficulty. Takeuchi et al. [20] showed that even though the task itself did not change, the pupil dilated if the demand level for the task increased, at least during the early phase of the learning process.

On the basis of these studies, we hypothesized that a relationship exists between pupil responses and SMEE induction. One popular hypothesis is that the misattribution process of perceptual fluency induces SMEE. Perceptual fluency is negatively correlated with the effort needed to analyze perceptual aspects. The amount of mental effort is reflected in the autonomic nervous response, including changes in pupil diameter. Thus, in this study, we examined the relationship between pupil response and SMEE strength.

Assessing the relationship between physiology and mere exposure is not new. In his classic paper, Zajonc [1] examined the relationship between the mere exposure effect (non-subliminal) and galvanic skin response (or skin conductance response, SCR). SCR refers to electrical conductance of the skin controlled by the sympathetic nervous system; SCRs are known to increase with mental effort [22]. Zajonc [1] showed that SCRs gradually decreased while participants repeatedly observed a visual pattern. Measurements were conducted while stimuli were presented supraliminally. Thus, questions regarding the relationship between the SMEE and autonomic nervous responses remain. Our specific hypothesis is that if perceptual fluency is the mechanism underlying the SMEE, pupil diameter should be less dilated for participants who exhibit the SMEE than for those who do not. To examine this hypothesis, we exposed participants to subthreshold visual stimuli while measuring pupil diameter with an infrared video-based eye-tracking device.

Kunst-Wilson and Zajonc [3] reported that the percentage of participants’ preference for exposed stimuli was about 60%. If a similar tendency is observed in our experiment, we can divide the data into two groups: one in which the SMEE is observed and one in which the SMEE is not observed. We can then compare the characteristics of pupil responses between the two groups to determine whether the pupil dilates less when the SMEE is observed than when it is not observed. In addition, we expected a similar relationship between pupil response and SMEE induction, even in within-participants data. As described later, we found that some participants exhibited the SMEE in some experimental sessions but not others, so we further sought to determine whether pupil responses differ between these sessions.

## Materials and Methods

### Ethics Statement

The study was reviewed and approved by the Research Ethic Committee of NTT Communication Science Laboratories. The nature and possible consequences of the experiments were explained to the subjects before the study began, and written consent was obtained from all subjects.

### Participants

Fourteen individuals (7 male and 7 female) aged 21 to 36 years (average age = 31.00 years, *SD* = 4.79 years) volunteered to take part in this experiment in exchange for monetary compensation of JPY 1,200 (US$ 11.00) per hour. Participants had no previous experience with psychophysical experiments and were naïve as to the purpose of the current experiment. All had normal or corrected-to-normal vision.

### Apparatus

Presentation of stimuli was controlled by MATLAB version 7.8 (MathWorks Inc.) with Psychophysics Toolbox version 3.0 extension [23], [24] on a personal computer (MacPro, Apple Inc.). Stimuli were presented on a 21″ RGB monitor (SONY GDM-F520). The monitor’s refresh rate was 120 Hz (therefore, the minimum presentation duration of any images was 8.3 ms), with a spatial resolution of 1024×768 pixels and 12-bit gray-level resolution. Average screen luminance for the monitor was set to 25.0 cd/m^2^. Participants observed the display with their head position maintained by a chin and head rest. Stimuli were viewed binocularly at a viewing distance of 57 cm. Pupil diameter of the right eye of each participant was recorded with a ViewPoint SceneCamera EyeFrames GigE Systems MSE07 (Arrington Research, Inc.) infrared eye-tracking device. The sampling rate of this eye tracker was 60 Hz. Synchronization of the visual pattern presentation on the CRT display and pupil data acquisition through the eye-tracking device was accomplished through an ethernet connection, “ViewPoint Eye Tracker” device control program, and Viewpoint Toolbox extensions (Arrington Research, Inc.) running on MATLAB. The experimental room was darkened and light shielded, with no other source of illumination present.

### Visual Patterns

**Figure 1:**
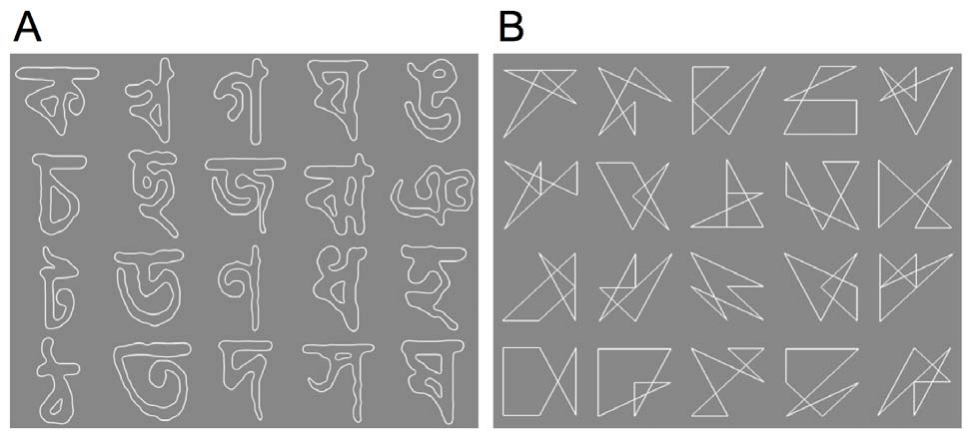
Visual patterns used in the experiment. (A) 20 Bengali characters. (B) 20 line drawings.

Two types of visual patterns were used: affect-neutral visual patterns, composed of Bengali characters, and line drawings. We prepared 20 patterns for each stimulus group as shown in Figure 1. Chinese characters have often been used in prior studies [1], [6]; however, we used Bengali characters because our participants were familiar with Chinese characters. Line drawing patterns were created by using an algorithm developed for this study that maintained the difference in total line length and number of corners between the different patterns within 10%. Each pattern subtended 12.0°×12.0° of visual angle. The background and the pattern were achromatic (x = 0.32, y = 0.29 in CIE1931 XYZ color space coordinates). The luminance was 25.0 cd/m^2^ for the background and 48.0 cd/m^2^ for the pattern. Thus, the luminance contrast of the stimuli was 31.5% in the Michelson relationship. This contrast value was determined based on a preliminary observation.

**Figure 2:**
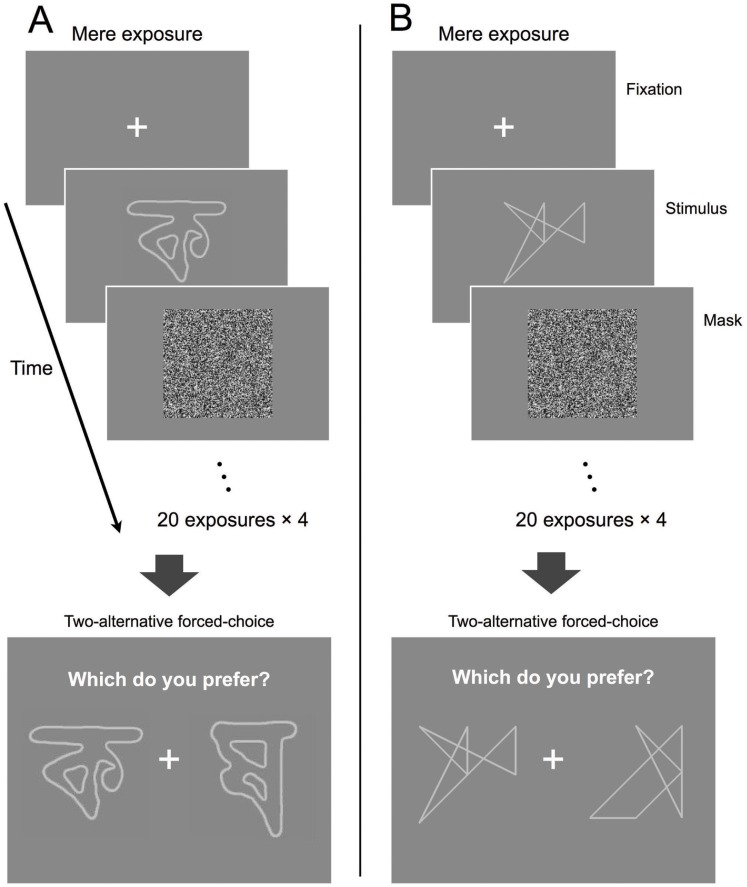
Schematic description of the experiment for the Bengali characters (A) and the line drawings (B). The fixation point was presented for 1(two exposures for 10 patterns). Each trial was repeated four times with a 30 s break between trials to constitute a single exposure session. After the exposure session, participants judged their preference by a two-alternative forced-choice task in which a pair of previously exposed (OLD) and unexposed (NEW) patterns was presented. The preference judgment task consisted of 80 comparisons.

Patterns were presented using a backward masking procedure; details are described in the next section. The masking stimulus was a random-noise pattern with the luminance of each pixel varying from 0 cd/m^2^ to 50 cd/m^2^ such that its average luminance was the same as that of the background. An example of the masking stimulus pattern is shown in Figure 2.

### Procedure

#### Mere exposure and preference judgments

Figure 2 shows a schematic description of the flow of the experimental session. The experimental design involved two phases: an exposure phase followed by a preference test phase. In the exposure phase, a white cross pattern fixation mark was presented in the center for 1 s. A visual pattern was exposed for 8.3 ms followed by a mask of 491.7 ms.

In a single exposure trial, 10 different visual patterns were presented twice, for a total of 20 exposures. Each exposure trial lasted 30 s: 20 exposures×(1.0 s for fixation +0.5 s for pattern and mask). Before each exposure trial, a blank screen with the same space-averaged luminance as the background (25.0 cd/m^2^) was presented for 500 ms to obtain pupil diameter baseline values. Details regarding pupil measurements are described below. During the exposure phase, participants were asked to try to concentrate (without blinking) on the center of the screen and avoid moving their eyes as much as possible. Having participants attend to visual patterns, even though they cannot be identified, is said to be important for inducing subliminal presentation effects [25].

The same trial, consisting of 20 exposures with the same presentation order, was repeated four times with a 30-s break between each trial. During the 30-s breaks, participants were instructed to relax but remain seated while continuing to look at the center of the display. Thus, a total of 80 exposures occurred before the preference test. We referred to this phase as the exposure session. Pupil diameter was measured while participants were exposed to the visual patterns and to the blank screen presented before the start of each exposure trial. Each participant was exposed to both types of patterns: Bengali characters and line drawings. As noted above, participants were exposed to 10 patterns of each type. We referred to the 10 patterns presented during the exposure phase as “OLD” stimuli. The total number of OLD stimuli was 20; 10 patterns made up of Bengali characters and 10 line drawings were randomly chosen for each participant. The two types of patterns were never mixed during the exposure phase. The other 10 patterns of each type were called “NEW” stimuli since participants were not exposed to them before the preference judgment test.

Following the exposure sessions, participants were asked to indicate their preference for one of the two presented stimuli, using a forced choice judgment task (Figure 2). The time between the final masking stimulus during the exposure phase and the presentation of a judgment choice display was 1 min. During this resting period, participants were asked to relax but remain seated. During the judgment task, a pair of stimuli comprising an OLD and a NEW stimulus was presented on the screen; participants selected the preferred stimulus by clicking a mouse. The preference judgment was made 80 times, during which 10 of the NEW and OLD patterns appeared eight times. Therefore, the number of supraliminal presentations of NEW and OLD stimuli was the same. The pairing of OLD and NEW stimuli was randomized for each participant. Each participant completed the experiments for both Bengali characters and line drawings. Half the participants were assigned to the Bengali character condition (Figure 2A) first and the line drawing condition (Figure 2B) second, while the other half were assigned to the line drawing condition first and the Bengali character condition second.

After completing the entire experiment, participants were asked to guess what they had seen during the exposure session and the nature of the experiment. None of the participants reported observing the mere exposure of the characters or line drawings.

#### Confirming the detectability of patterns

We checked the detectability of the visual patterns after the main experiment described above. In the experiment by Kunst-Wilson and Zajonc [3], participants were asked different types of questions concurrently after the exposure session: a detection task to determine whether the pattern was recognized, and an affective judgment task to evaluate participants’ preferences. In our experiment, we did not want participants to guess the content of presented patterns during exposure, since this guessing might have an unpredictable influence on pupil responses. Therefore, we did not concurrently run the experiment to check whether the pattern was detectable during the main experiment shown in Figure 2.

We used a two-alternative, temporal forced-choice procedure. In one of the two intervals, only the mask pattern was presented. In the other interval, both the mask and the pattern (Bengali character or line drawing) were presented. In this backward masking procedure, the pattern was presented for 8.3 ms followed by the mask and presented for 491.7 ms, similar to what is shown in Figure 2. Thus, the total duration was 500 ms. When the mask was presented separately, its presentation duration was 500 ms. The participant, by pressing one of two buttons, indicated which interval contained the visual pattern rather than the mask. The two intervals were separated by a 1-s gray-colored blank field of average luminance ( = 25 cd/m^2^ as in the main experiment); the onset of each interval was marked by an auditory cue. No feedback was given. We randomly selected 10 Bengali characters and 10 line drawings for each participant. Each pattern was presented 10 times; hence, each participant judged a total of 200 visual patterns ( = (10+10)×10). The pattern presentation order was randomized for each participant.

The average percentage of correct responses for all participants was 52.4% for the Bengali characters and 51.1% for the line drawings. Results were not significantly different from chance for Bengali characters (*t*(13) = 0.16, *p = *0.87, *n.s.*) or line drawings (*t*(13) = 0.37, *p = *0.72, *n.s.*). Thus, we can confirm that every backward-masked, 8.3-ms presentation of a Bengali character or a line drawing was below participants’ detection threshold.

## Results

We confirmed that the presented visual patterns (Figure 1) were below the detection threshold. Therefore, if we found an effect similar to that reported by Kunst-Wilson and Zajonc [3] in the forced-choice preference task subsequent to mere exposure (Figure 2), we would observe a subliminal mere exposure effect (SMEE). Due to an error with the eye-tracking device, we removed the entire dataset of one participant from the following data analysis.

**Figure 3:**
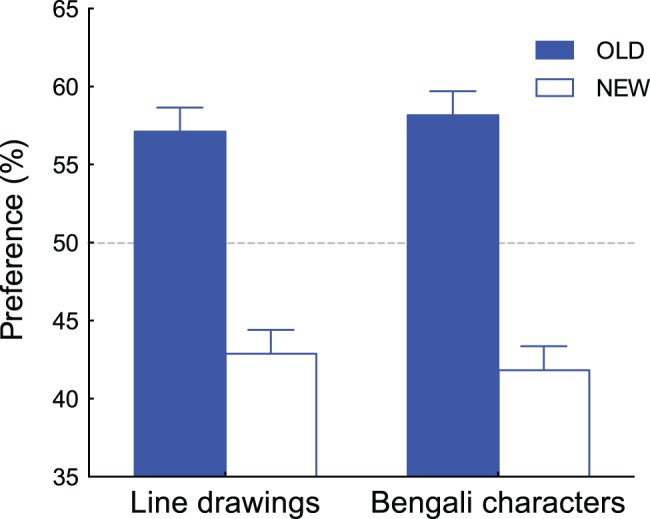
Results of the forced-choice preference task. Percent preferences of the OLD and NEW stimuli are plotted for Bengali characters and line drawings, respectively. Error bars represent ±1 SEM.

Results of the forced-choice preference task are shown in Figure 3. Since 13 participants completed 160 comparisons during the preference judgment task (80 for Bengali characters and 80 for line drawings), 2,080 comparisons in total were analyzed (1,040 for Bengali characters and 1,040 for line drawings). We found that the OLD stimuli were preferred 605 times out of 1,040 (58.2%) for Bengali characters and 594 times (57.1%) for line drawings. Both percentages were significantly higher than a chance preference of 50% (*t*(12) = 2.45, *p*<0.05, Cohen’s *d* = 0.68 for Bengali characters; *t*(12) = 2.21, *p*<0.05, Cohen’s *d* = 0.61 for line drawings). These results are comparable to those obtained by Kunst-Wilson and Zajonc [3]. We also found no difference between preference percentages for Bengali characters and line drawings (*t*(12) = 0.22, *n.s.*). Therefore, we analyzed the combined data of both patterns for the pupil response analysis.

**Figure 4:**
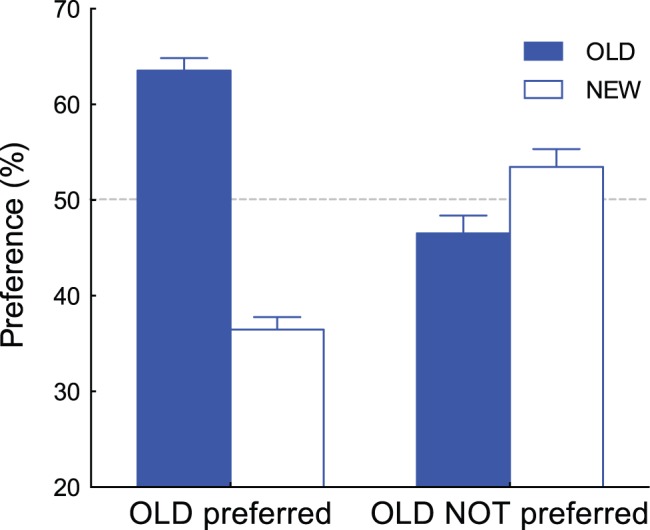
Percent preferences of the OLD and NEW stimuli for the “OLD preferred” group (n = 17) and “OLD NOT preferred” group (n = 9). Error bars represent ±1 SEM.

Although averaged data confirmed the existence of the SMEE, we found that the effect was not observed for all experimental sessions. To analyze pupil responses, we divided the sessions into two groups: one where the majority of comparisons favored the OLD patterns and sessions where NEW patterns were favored. Since each participant completed two sessions involving two different patterns (Bengali characters and line drawings), we had data sets for 26 sessions. Each session contained 80 comparisons, so we calculated how many times OLD stimuli were preferred out of 80 comparisons. We grouped the session as “OLD preferred” if the resulting number of comparisons exceeded 40 (half of 80). A tendency to prefer OLD stimuli was observed in two thirds of the datasets (17/26). In the other datasets (9/26), preference for the NEW stimuli was higher than or identical to that for OLD stimuli. We defined the first datasets (*n* = 17) as the “OLD preferred” session group and the second datasets (*n* = 9) as the “OLD NOT preferred” session group. Figure 4 shows the percentage of preferences for OLD and NEW stimuli for each group. In the OLD preferred group, the OLD stimuli were judged as preferred (63.5%) significantly above chance (*t*(16) = 5.63, *p*<0.0001, Cohen’s *d* = 1.37) but not for the OLD NOT preferred group; rather, the NEW stimuli were judged as preferred (53.5%) significantly above chance (*t*(8) = 3.13, *p*<0.05, Cohen’s *d* = 1.04).

**Figure 5:**
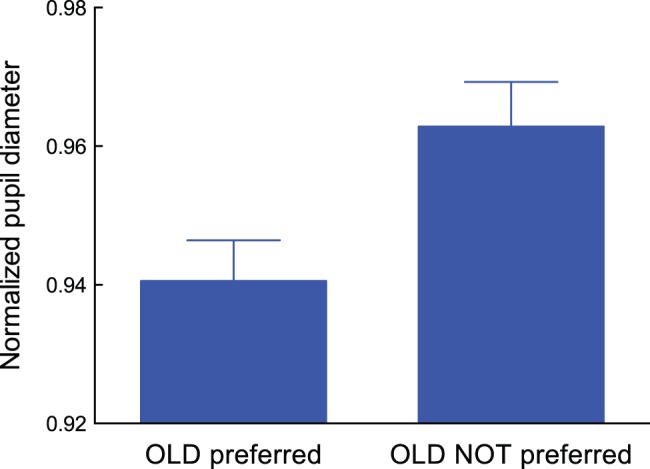
Normalized pupil diameter during subliminal mere exposure for the averaged dataset of the “OLD preferred” group (n = 17) and the averaged dataset of the “OLD NOT preferred” group (n = 9). Error bars represent ±1 SEM.

Our main question of interest was whether pupil responses at the mere exposure phase differed between groups. To quantitatively evaluate pupil diameter during exposure, we performed the following calculations. First, we normalized pupil diameters by using the mean pupil diameter during the 500-ms blank screen presentation before the beginning of the four exposure trials. Therefore, the pupil diameters recorded at each exposure trial were normalized separately. No signal smoothing was applied. Eye blinks were detected by the ViewPoint Eye Tracker software (see “Materials and Methods” section) and removed from the raw data. Then, missing data points were reconstructed off-line using a standard spline interpolation. We next averaged the normalized diameter of the pupil during exposure separately for each group. The results were then plotted in Figure 5.

An unpaired *t*-test shows that average pupil diameter during mere exposure was smaller for the “OLD preferred” group than for the “OLD NOT preferred” group (*t*(24) = 2.39, *p*<0.05, Cohen’s *d* = 1.38). This result, shown in Figure 5, is consistent with our speculation regarding the relationship between pupil constriction and SMEE induction.

Our hypothesis would be strengthened if a similar relationship were found for within-participant data in addition to the between-participant data. From the preference judgment test data, we discovered that five participants exhibited a different tendency with regard to pattern type: two exhibited preferences for OLD stimuli among the Bengali characters but not for the line drawings, while three participants showed the opposite preference pattern. We are unsure about why this stimulus dependency was observed for some participants; future research may need to examine individual differences in the SMEE. Nonetheless, analysis of these participants’ pupil data could reveal whether the tendency shown in Figure 5 can be observed in within-participant data.

**Figure 6:**
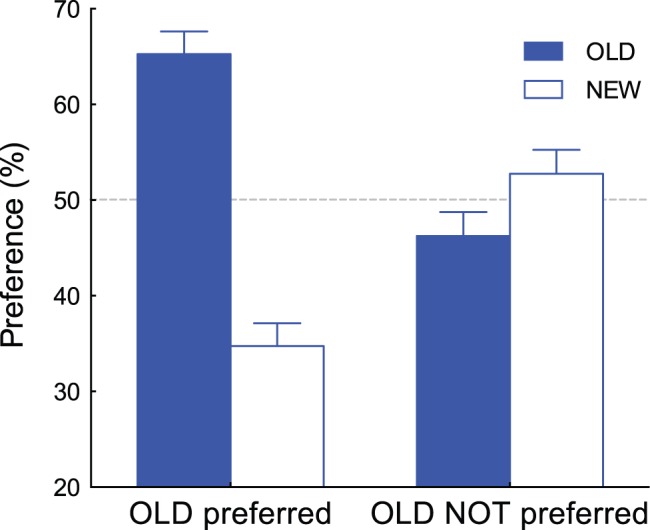
Percent preferences of the OLD and NEW stimuli for the “OLD preferred” group and “OLD NOT preferred” group. Data from 5 participants who showed a different tendency to the two types of patterns are presented. Error bars represent ±1 SEM.

Similar to the calculations represented in Figure 4, the datasets of the five participants for the two types of patterns were divided into two groups (“OLD preferred” and “OLD NOT preferred”). For the five participants, the preference percentages for the OLD and NEW stimuli for each group are shown in Figure 6. A similar tendency shown in Figure 4 was observed within subjects; the OLD stimuli were judged as preferred (65.3%) significantly above chance (*t*(4) = 3.16, *p*<0.05, Cohen’s *d* = 1.41) in the OLD preferred group, whereas the NEW stimuli were judged as preferred (52.8%) significantly above chance (*t*(4) = 2.99, *p*<0.05, Cohen’s *d* = 1.34) in the OLD NOT preferred group.

**Figure 7:**
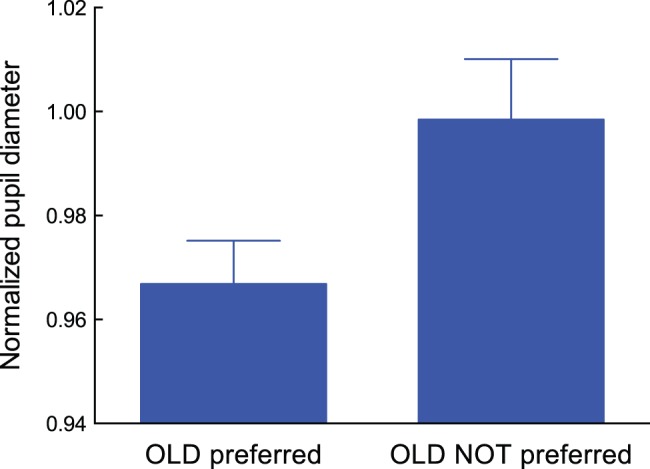
Normalized pupil diameter during subliminal mere exposure for the averaged dataset of the “OLD preferred” group and the averaged dataset of the “OLD NOT preferred” group. Data from 5 participants who showed a different tendency to the two types of patterns are presented. Error bars represent ±1 SEM.

Similar to the data analysis depicted in Figure 5, we also calculated and plotted the five subjects’ normalized pupil diameters during exposures for each group (Figure 7). Each bar represents the average value. We should note that data from the same participant is included in each of these results. A paired *t*-test shows that pupil dilation was less prominent for participants who exhibited a preference for OLD stimuli rather than for NEW stimuli during the preference judgment task (*t*(4) = 4.63, *p*<0.01, Cohen’s *d* = 1.00). This further supports our speculation regarding the relationship between pupil responses and SMEE induction.

Pupil response is modulated by patterned stimuli that do not change overall ambient light levels [8], [26], [27]. During pupil measurement, we presented different types of visual stimuli during a single exposure: a fixation mark, a visual pattern (Bengali characters or line drawings), and a masking pattern (Figure 2). These stimuli provoked a pupil reflex; a typical consequence is rapid pupil constriction after the onset of the visual stimulus. The next question is whether a similar pupil reflex would be observed between the “OLD preferred” and the “OLD NOT preferred” group, even when there is a difference in the time-averaged pupil diameter between the two groups, as shown in Figure 7.

**Figure 8:**
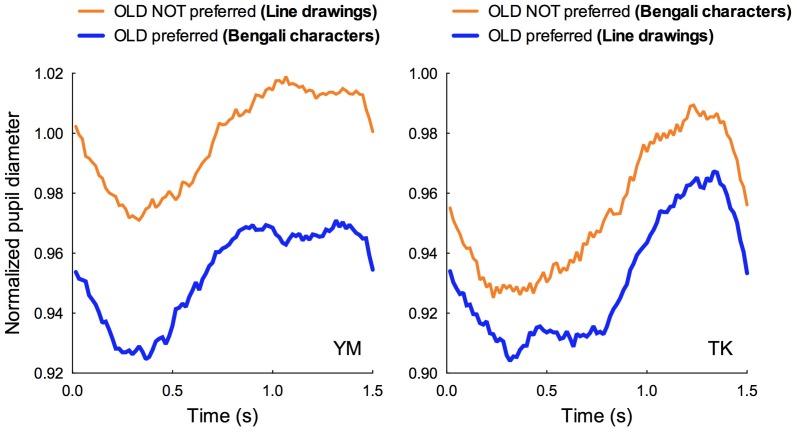
Normalized pupil diameter during a single exposure session as a function of time. Pupil diameter was averaged over 80 exposures (20 exposures×4, as shown in Figure 2). The fixation mark was presented from 0.0 to 1.0 s on the horizontal axis. The visual pattern and the mask pattern were presented following the 0.5-s period. The blue function denotes the pupil diameter obtained from the “OLD preferred” group while the orange function denotes the pupil diameter obtained from the “OLD NOT preferred” group. Left graph: data from participant YM. YM exhibited preferences for OLD stimuli among Bengali characters but not for the line drawings. Right graph: data from participant TK. TK exhibited preferences for OLD stimuli among the line drawings but not for the Bengali characters.

Figure 8 shows the time-varying pupil diameters averaged from 80 exposures (see Figure 2) of the “OLD preferred” and the “OLD NOT preferred” groups from two participants: YM and TK. YM exhibited preferences for OLD stimuli among Bengali characters but not for the line drawings. On the other hand, TK exhibited preferences for OLD stimuli among the line drawings but not for the Bengali characters. On the horizontal axis of the Figures, the fixation mark appeared at 0.0 s and continued for 1.0 s. The visual pattern and mask pattern appeared at 0.5 s.

As shown in Figure 8, within each participant, the shape of the two functions are quite similar. This indicates that Bengali characters and line drawings induced an equivalent pupil reflex. However, two functions are separated along the vertical axis for both participants, which indicates that the time-averaged pupil diameter is smaller in the “OLD preferred” group than in the “OLD NOT preferred” group. Since participants other than YM and TK showed a similar tendency as displayed in Figure 8, we assumed that mental effort modulated by subliminal mere exposure was responsible for differences in time-averaged pupil diameter (shown in Figure 7).

## Discussion

In the current study, participants observed affect-neutral stimuli in a task using a backward-masking paradigm. Following multiple stimulus presentations, participants performed a forced-choice preference judgment task intended to assess preferences for OLD versus NEW stimuli. In general, those receiving subliminal stimuli preferred OLD stimuli, indicating an SMEE effect. During the exposure phase, we found less pupil dilation among participants who exhibited a preference for OLD stimuli throughout a series of exposures than among those who did not show this preference. A similar relationship was observed in both between- and within-participants data. Furthermore, by examining within-participant time-varying pupil diameters, it is possible that pupil reflex components are not playing a decisive role.

As shown in Figures 7 and 8, normalized pupil diameter was close to 1.0 for the “OLD NOT preferred” group while pupil diameter was smaller than 1.0 for the “OLD preferred” group. As shown in Figure 8, the function obtained from the “OLD preferred” group shifts downward while its shape was nearly equivalent as that obtained from the “OLD NOT preferred” group. This indicates that less pupil dilation is critical for exhibiting preference toward a repeatedly presented pattern. This further supports our assumption that a decrease in mental effort accompanied by less pupil dilation is important for provoking the SMEE.

We should mention that our results seem to be consistent with other hypotheses proposed so far. Zajonc [28], based on classical conditioning theory, suggested that a stimulus presented repeatedly without any aversive event produces a positive feeling by reducing arousal related to any feelings of aversion or anxiety. The uncertainty reduction model [2], [29] also assumes that a decline in arousal is a key factor for enhancing preferences to exposed stimuli. Meanwhile, there has been some dispute regarding which aspect the pupil response represents: hedonic valence or emotional arousal [30]–[32]. A recent study by Bradley et al. [33] showed that pupil diameter is determined by emotional arousal not hedonic valence. They found that pupillary changes were greater when participants viewed emotionally arousing pictures, regardless of whether these were pleasant (high valence level) or unpleasant (low valence level). Based on this study, the data shown in Figures 5 and 7 indicate that when the SMEE was induced, participants’ arousal decreased more as compared to when the SMEE was not observed. Our results, therefore, seem to be consistent with the prediction from models that assume participants’ arousal is the key factor for inducing the SMEE. However, we speculate that perceptual fluency and arousal are not contradictory but related. Thus, the hypotheses based on perceptual fluency and arousal might capture different aspects of the SMEE. Further research is needed to clarify this point.

Finally, neurophysiological aspects of pupil responses regarding SMEE need to be addressed. The system controlling pupil responses is extremely complex. Pupil dilation is controlled by the sympathetic nervous system, while its constriction is controlled by the parasympathetic nervous system. Pupil constriction is attained either by an activation of the pathway including the Edinger-Westphal nuclei in the midbrain or by an inhibition of the pathway including the posterior hypothalamic nucleus [34]. Further, neurophysiological studies have shown that neurons in various subcortical and cortical regions appear to send signals to control pupil diameter [8]. Findings on the pupil light reflex, which has been studied for decades, are contradictory. A recent study shows that pupils will constrict when observers feel illusory brightness enhancement, even when the luminance remains physically the same [35]. This study indicates that pupil response reflects mental states related to visual illusion; thus, the inclusion of higher visual cortices is required. Further, pupil responses are shown to reflect various kinds of cognitive control related to attention, though this mechanism is still under examination [36]–[40]. Our results indicate that pupil responses can be considered a “window into the mind” even in a task where conscious perception is not required. A subcortical and cortical level of examination would be needed to understand the nature of pupil responses during a preference judgment task. This is an exciting direction for future research in order to understand how and why we prefer one thing to another and how our decisions may be related to psychophysiological processes.
